# Technology matters: AI‐driven tools in children's mental healthcare: perspectives from young people and parents

**DOI:** 10.1111/camh.70045

**Published:** 2025-11-12

**Authors:** Zoë Firth, Christine Aicardi, Stephen Oram, Nicholas Cummins, Alice Wickersham, Helen L. Fisher, Johnny Downs

**Affiliations:** ^1^ CAMHS Digital Lab, Department of Child and Adolescent Psychiatry, Institute of Psychiatry, Psychology and Neuroscience King's College London London UK; ^2^ Department of Global Health and Social Medicine King's College London London UK; ^3^ Independent Applied Science Fiction Practitioner London UK; ^4^ King's Institute for Artificial Intelligence King's College London London UK; ^5^ Department of Biostatistics and Health Informatics, Institute of Psychiatry, Psychology and Neuroscience King's College London London UK; ^6^ Social, Genetic and Developmental Psychiatry Centre, Institute of Psychiatry, Psychology and Neuroscience King's College London London UK; ^7^ ESRC Centre for Society and Mental Health King's College London London UK; ^8^ South London and Maudsley NHS Foundation Trust London UK

**Keywords:** Adolescence, prediction, parent–child relationships, early intervention, communication

## Abstract

Artificial intelligence may be able to improve the efficiency, accuracy and predictiveness of mental health assessments, and public perspectives are crucial to ensuring these tools are implemented with fairness and accountability. We developed an artificial intelligence‐driven tool to automatically analyse parents' speech data, which if successful could be implemented in children and young people's mental health assessments. To engage stakeholders with this project, we worked with a science fiction writer to produce two stories about possible futures of our tool, which we discussed in workshops with young people and parents. Here, we summarise key themes arising from this novel method of engaging the public in mental health research, and highlight considerations for clinicians and researchers creating novel technologies for children and young people's mental healthcare.

**Key Practitioner Message**

**What is currently known?**

- Tools driven by artificial intelligence may be able to help predict the risk of mental health disorders in children and young people, but input from the public is needed to develop these tools responsibly.

**What has been shown?**

- We used two science fiction stories about our proposed artificial intelligence‐driven tool as the basis for engagement workshops with young people and parents.

**What is the significance for clinical practice?**

- Some implementations of AI‐driven technology in children's mental healthcare may be received favourably, but young people and parents are keenly aware of challenges such as the socioeconomic accessibility of these technologies and the impact and utility of predicting mental health outcomes.

## Background

Artificial intelligence (AI)‐driven technologies show great potential to streamline and improve the assessment and treatment of mental health disorders. Applications of AI‐driven language analysis tools in the assessment of mental health include the use of affective computing to detect emotion in speech (Wang et al., 2022) and the identification of vocal biomarkers (Fagherazzi, Fischer, Ismael, & Despotovic, 2021). Within this field, our work has been focused on developing AI‐driven tools to assess expressed emotion in mothers' speech about their children (Mirheidari et al., 2024). Expressed emotion is used to assess ‘the emotional climate of the parent–child relationship’, and levels of parental warmth and negativity are often measured using the Five‐Minute Speech Sample (FMSS) during which a parent speaks freely about their child for five minutes (Sher‐Censor, 2015). While measures of parental expressed emotion have predictive validity for children's future mental health outcomes, indicating these measures could be used to identify children at risk of mental health disorders and thus target preventative intervention (Peris & Miklowitz, 2015), it is currently too resource‐intense for clinicians to apply and measure in clinical settings. Therefore, we wanted to explore whether an AI‐driven tool could be trained to classify expressed emotion from the FMSS.

Acquiring input from members of the target population is essential in the development of any novel clinical tool, especially when investigating potentially contentious tools such as AI‐driven and predictive technologies. Given the abstract nature of the research, we employed a novel method of public engagement, collaborating with an author experienced in writing applied science fiction (stephenoram.net/science‐and‐scifi‐projects) to write two short stories on possible futures of the proposed clinical tool (Oram, 2023). Appendix S1 ‘A Mother's Nightmare’ tells the story of a mother undergoing a FMSS assessment to get support for her daughter, while Appendix S2 ‘Standard Deviations’ is about a young mother who must disclose her mental health risk score from childhood to apply for a job. We read these stories in separate workshops with six parents and 12 young people to initiate conversations about the implications of our research. Participating parents included autistic and nonautistic parents of autistic children. Young people were aged 13–18; both young people's groups we consulted (see ‘Acknowledgements’) intentionally recruit from diverse sociodemographic and ethnic backgrounds.

## Parents' perspectives

“AI should never be more than a helper for grunt work, not making life‐changing decisions.”

“We already know what causes poor life outcomes. Focus on making society equal and these things will largely be a non‐issue, rather than allowing inequality to persist, and intervening when you predict it might harm someone… that applies regardless of whether it's a human or AI involved.”

### Limitations of AI‐driven tools

Parents raised several concerns over the potential for bias in the proposed tool, including *bias against neurodivergent parents* who may communicate differently. They were sceptical that an AI‐driven tool would be sufficiently sophisticated to interpret the *complexity and nuance of human communication*. Nevertheless, parents spontaneously suggested positive applications of AI‐driven tools in healthcare, including its use to *streamline the initial stages of mental health and neurodiversity assessments*, or to *synthesise diagnostic information* for clinicians and/or parents.

### FMSS and the complexity of parenting

Parents stressed how focusing on negative emotion could entrench *parent‐blaming attitudes to children's mental health*. They noted the risk that parents, especially those navigating a healthcare system with years‐long waiting lists, might give an inaccurate account of their child to ‘game the system’ for enhanced treatment access. Parents also highlighted how parenting varies substantially on a daily basis as well as across the life span, and therefore expressed doubts about the ability of a single FMSS, whether coded by a human or technology, to capture the *complexity of parenting*. Parents outlined the limitations and variable accuracy of predicting mental health outcomes, and raised the possibility of predictions having a ‘self‐fulfilling prophecy’ effect on a child's development.

### Data and privacy

Parents responded strongly to the narrative theme that people's access to services could be contingent on them providing personal data. They raised broader concerns about the government's use of health data and the importance of *dynamic, continuous models of consent*. One parent raised the issue that while our use of the FMSS was fully anonymised, future AI technology could potentially de‐anonymise families.

### Assessment versus treatment

Overall, parents emphasised that the focus on increasing resource efficiency with the proposed AI‐driven tool belied core issues in children's mental healthcare which an automated speech analysis tool would not meet, namely *timely access to clinical expertise and evidence‐based treatment*. Parents noted they had been given very different information by different clinicians on their children's mental health and neurodivergent diagnoses, acknowledging the potential for clinicians to perpetuate bias. They also highlighted concerns over *treatment accessibility* and the widening gap between public and private healthcare.

## Young people's perspectives

### Strong preference for human doctors

Multiple young people highlighted that doctors are specifically trained for the purposes of mental health assessments and diagnosis, with one young person raising concerns about the ability of AI‐driven tools to account for complex mental health issues that are more difficult to categorise. They expressed similar concerns to parents regarding the ability of AI to capture nuances in communication; overall, young people named fewer perceived benefits of the tool than parents. Nevertheless, many young people agreed that having access to the tool would be better than nothing, and had multiple suggestions for improving its implementation, such as capturing children's speech samples in addition to parents' samples.

### Accuracy of the FMSS

Like parents, young people also outlined the limitations of the FMSS in assessing a child's family environment. They raised concerns over the accuracy of some parents' accounts of their children, due to reasons such as not wanting to be negatively judged, not knowing their child's emotional welfare, or not coming from a family or culture where mental health is discussed. Also like the parents, young people raised *concerns over the impact of receiving a prediction of future mental health disorder* on a child's development, both the potential ‘self‐fulfilling prophecy’ effect and the possibility that parents might treat their children differently.

### Data and accessibility

Concerns around whether everyone would have access to the tool were highlighted. Young people wanted to know who would have access to their data and for how long, and expressed concerns that their data could be made public or de‐anonymised. They also asked where the AI‐driven tool got its training data, what coding language we were using and whether it is secure, illustrating their engagement with the technical aspects of the project.

## Implications

Our discussions with parents and young people raised several key considerations for clinicians and researchers working to develop novel technologies for children's mental healthcare.

Young people and parents were highly aware of and engaged with the potential risks of implementing AI‐driven technology in children's mental healthcare. All groups highlighted concerns around *data security and privacy*, the *potential for bias* and whether AI‐driven tools would be able to *capture nuance in human communication*. Many young people explicitly expressed a preference for humans over an AI‐driven tool performing the FMSS assessment, highlighting concerns around transparency; indeed, any lack of interpretability within *the ‘black box’ of AI functionality* may be a critical barrier to address in the development of AI‐driven clinical tools.

Nevertheless, both parent groups gave spontaneous suggestions for applications of AI‐driven tools in healthcare, highlighting its potential to improve information accessibility, waiting lists and the objectivity of assessments. This finding accords with a recent public dialogue report commissioned by NHS AI Lab (Ipsos, 2022), where participants also gave spontaneous suggestions for implementations of AI‐driven technology in healthcare. When developing a novel clinical technology, it is therefore important to capture the *broad spectrum of public attitudes*, not assuming they will be negative.

Overall, the dominant themes focused less on the inherent issue of using AI‐driven technology in healthcare, and more on the concerns around its implementation, as well as the validity of the FMSS assessment. *The sociotechnical implementation of novel technologies in children's mental healthcare is a key public concern*. Young people and parents were keenly aware of discrepancies in the availability of timely assessment and treatment across private and public mental healthcare, and asked multiple questions about how access to the tool would be regulated. In a time of widening health inequalities and acute mental health service burden, the participants' concerns with sociodemographic accessibility illustrate how implementation is an essential consideration from the early stages of clinical technology development. Participants also outlined multiple *concerns with predicting children's future mental health* with assessments like the FMSS, including doubts about the utility of mental health predictions compared to timely, responsive and personalised treatment. As research in preventative medicine often concerns developing tools that can predict mental health or treatment outcomes, clinicians and researchers must work to ensure any predictive tools that are translated into children's mental healthcare involve rigorous consultation with the public.

Finally, *storytelling is a productive method of engaging the public in collective decision‐making on novel technologies*. Using storytelling to stimulate discussions around technology gives young people and parents the opportunity to engage in clinical research not only in their capacity as potential service users, but also as digital citizens of a world where the deployment of AI‐driven technology will continue to increase in the coming years. The nuance and breadth of participants' insights generated by the stories, including on technical aspects of the tool, indicate that factual or technical descriptions of a technology are not the only types of information that breed understanding.

## Conclusion

Attitudes to AI‐driven tools in children's mental healthcare are varied; it should not be assumed that all views will be negative. Our consultations with young people and parents highlighted their concerns around the sociotechnical implementation of such tools, underscoring the need for public engagement at every stage of health technology development and research.

## Conflict of interest statement

The authors have declared that they have no competing or potential conflicts of interest.

## Funding information

The work described was funded by the Medical Research Council (MRC) [MR/X002721/1] and the Psychiatry Research Trust [39C]. A.W. was supported by ADR UK (Administrative Data Research UK), an Economic and Social Research Council (ESRC) investment (part of UK Research and Innovation) [ES/W002531/1]. N.C. is part funded by the National Institute of Health and Care Research (NIHR) Maudsley Biomedical Research Centre at South London and Maudsley NHS Foundation Trust and King's College London and supported by the NIHR University College London Hospitals Biomedical Research Centre. H.L.F. receives salary support from the ESRC Centre for Society and Mental Health at King's College London [ES/S012567/1]. J.D. received support from a NIHR Clinician Scientist Fellowship [CS‐2018‐18‐ST2‐014]. For the purpose of Open Access, the authors have applied a CC BY public copyright licence to any Author Accepted Manuscript version arising from this submission. The views expressed are those of the authors and not necessarily those of the ESRC, MRC, NIHR, Department of Health and Social Care, or King's College London. The funders had no role in the study design, data collection, analysis, interpretation or writing of the report.

## Ethics statement

The described workshops with parents and young people fell under the Patient and Public Involvement and Engagement (PPIE) activities of the project and therefore required no ethical approval.

## Supporting information


**Appendix S1.** A Mother's Nightmare.

**Appendix S2.** Standard Deviations.

## Data Availability

Data sharing is not applicable to this article as no datasets were generated or analysed during the current study.
