# Supplementary material for: Technology matters: AI‐driven tools in children's mental healthcare: perspectives from young people and parents
Source: Child Adolesc Ment Health. 2025 Nov 12;31(3):244–7. doi: 10.1111/camh.70045 (PMC13474275; doi:10.1111/camh.70045)
Supplement: Supplementary file 1 [file CAMH-31-244-s001.docx]

First published in Extracting Humanity and Other Stories by Stephen Oram, Orchid’s Lantern, 2023

**Technology Matters: AI-driven tools in children’s mental healthcare: perspectives from young people and parents**

**Supporting Information**

**Appendix S1**

A Mother’s Nightmare

Stephen Oram

The room smelt of people. I’m not kidding you. That, and polished wood. It was nice. Comforting. Not like those NHS rooms, all sterilised and automated with no human being in sight. The doctor was nice too. A psychiatrist. I liked her. A bit stiff in the way she spoke to me, but you could hear the kindness behind it. I got the feeling she cared. You know, properly cared. It put me at ease. It was what I needed. It wasn’t every day my words would have such an effect on my daughter’s future. I have to be honest, though—I was worried, and I know how I get when I’m worried. Flummoxed. Can’t help it, really. I blame it on my mother. Always urging me to do more. To be better. I guess I have to thank her, though. Not for that, of course, but for her savings. All that money she’d saved up to go on the holiday of a lifetime. I hope I did right using it for Rosie’s mental health prediction. Not that my mother cares. How can she when she’s a pile of ashes? Sorry, that was uncalled for.

As well as smelling of people and wood, the room was kitted out with all sorts of gadgetry on the walls and hanging from the ceiling. Cameras, mostly. Those heat-sensing ones they use at airports. The ones that check if you’re lying. I’m told they can see under your skin. That they can detect all sorts of emotions you’d rather keep hidden. What a world we’ve made for ourselves.

When I arrived, the doctor was sitting in a low comfy chair, smiling.

“Hello,” she said. “Ms Banbury, isn’t it?”

I smiled back. “Janelle. Jan.”

“So, you want to know how much private health care would cost for—” She glanced at the screen on her lap. “Rosie.”

“Yes. I need a prediction of her future mental health. As part of the financial assessment.” She waggled a finger at one of the cameras mounted on the wall above her. “Are you happy for the artificial intelligence to begin recording as a backup to me?” I wasn’t, but I don’t think I had a choice.

“No problem,” I said.

“Tell me about Rosie.”

I took a big breath, knowing the next five minutes would be crucial. I was desperate to remember word for word what I’d practised with my friends, but I couldn’t get a grip on how we’d decided I should start. I said something like she was great at drawing and a gifted drummer.

I may also have said that she was a bit of a maths genius. A small white lie. Then I dried up.

Trying too hard, I guess.

The doctor let the silence hang there for a while—probably only a few seconds, but it seemed like hours—until she prompted me with that soft, warm voice of authority.

“Let me help. What was she like this morning?”

I sighed. Mornings were always the most difficult. She didn’t like getting out of bed. Even for something she was looking forward to. Five years old and already I could see my dad in her.

“Mornings?” I said and grinned. “She’s a nightmare.”

She didn’t see the grin. Too busy looking out of the window at the rain.

“I see,” she said in an irritated, distracted kind of way.

Her screen lit up and she lifted it off her lap. I stopped talking and waited for her to say something. She was busy reading and tutting. Eventually, she looked at me and spoke.

“Whatever I may think, it would seem that I am in danger of bringing my emotions into this situation. The AI will take over proceedings.”

“But—”

She looked away. “‘She’s a nightmare.’ A favourite of my mother’s. Please continue.”

“What am I paying for if the AI is going to do it? I could have done that on the NHS for a lot less money. I want you to carry on.”

“You don’t,” she said. “Believe me.”

I hesitated. Wishing the decision wasn’t mine to take alone. Sole carer, or as good as. Should I carry on? I had no choice. I started to talk about Rosie. So far off-script by now that I wasn’t even thinking about what I was saying. Just telling stories about her. Remembering the good times. Explaining the bad. It seemed like a long time before the doctor spoke again, to thank me for being so open and honest. Apparently, it had only been five minutes.

She consulted her screen before speaking.

“My faithful AI here concludes that there’s a sixty per cent chance that Rosie will develop some issues later in life. And that its prediction is accurate to around seventy-three per cent.”

I was flabbergasted. I’d expected something more rounded. More verbal. Less number crunching.

“What do you think? Do you agree with it?” I asked.

“To be honest, most of the time it’s better at this stuff than me.”

“But I’m here with you. We’re talking. Rosie isn’t a statistic.”

“True, but I’m biased. Although it’s also true that I can spot the anomalies better than it can. I can deal with complexity much better, too. I don’t have to categorise everything.” She hesitated as if deciding what to say next.

“Despite my abilities, it has access to a lot more experience than I do, and when you’ve gone it’ll give me a training session. To improve my performance.” I pulled a face. I couldn’t help it.

“Why on earth don’t you work together?”

She ignored me and handed me the screen.

“There’s the cost,” she said. “Based on Rosie’s physical condition and all the other data we hold,” she quickly added.

Two sets of numbers were displayed. The average healthcare package, which came in at seventeen per cent of earnings — five per cent above the NHS — and Rosie’s at thirty-nine per cent. Even by claiming the National Insurance back, which I could if I was to go private, this was way out of my reach. The doctor seemed to sense my despair.

“There is an alternative,” she said.

“Go on.”

She told me all about a trial that Rosie could join. Her data would be gathered and sold to the NHS. They needed to increase the diversity of their training data for their version of the predictive AI, and for as long as Rosie was on the trial, she’d get her private healthcare at the average rate of seventeen per cent. I have to say, it seemed like a win–win to me, so I agreed right there and then. The doctor began the process of uploading Rosie’s data into the trial. She gave me a running commentary about how Rosie really fit the bill. She was the right sex, gender, and ethnicity. With a grin, the doctor confirmed that we were in one of the least represented socio-economic groups.

It was all going well.

Until.

“There’s one part of the data we need to complete for full acceptance into the trial.”

“Which is?”

“We need a five-minute sample of Rosie’s father speaking about her. Won’t take long to organise and then you’re good to go.”

And there it was. Again. Without a father, Rosie was excluded. It didn’t matter why her father wasn’t to hand, and I certainly wasn’t going to tell them.

“She doesn’t have one,” I said.

The doctor folded her arms and sighed.

She seemed to be collecting her thoughts and then, in a soft voice, she spoke.

“I am so sorry for you both,” she said.
