# Supplementary material for: Technology matters: AI‐driven tools in children's mental healthcare: perspectives from young people and parents
Source: Child Adolesc Ment Health. 2025 Nov 12;31(3):244–7. doi: 10.1111/camh.70045 (PMC13474275; doi:10.1111/camh.70045)
Supplement: Supplementary file 2 [file CAMH-31-244-s002.docx]

First published in Extracting Humanity and Other Stories by Stephen Oram, Orchid’s Lantern, 2023

**Technology Matters: AI-driven tools in children’s mental healthcare: perspectives from young people and parents**

**Supporting Information**

**Appendix S2**

Standard Deviations

Stephen Oram

Malcolm is my favourite cousin. The one who’s always at my side when I need someone. Today is no exception. He tickles my gorgeous baby Sofia under her chin and sits down.

“Here, read this,” I say and pass him the response to my application.

I swallow, trying to get rid of the lump in my throat. They’ve turned me down. My dream destroyed by an automated message. How could they?

I was twelve when my sister was arrested and I decided I had to become a police officer. Fair enough, she was an entitled brat who did what she wanted, but it was police prejudice at play for certain and she was lucky to escape prison. It made me want to change things from the inside.

While Malc dutifully reads, I bounce Sofia up and down on my lap and watch the wispy clouds drift across the sky. I savour the fragrance of freshly cut grass wafting up from the lawn. Try to think about something else. My parents and their hedonistic friends are raucous. As chaotic as ever. They have highly paid jobs and they party hard. Today’s excuse—my eighteenth birthday. The doorway to adulthood. Supposedly.

“Oh,” he says. “I get it.”

“What?”

“What it says. You’re difficult to place.” He chuckles. “You are kind of a one-off. You’ve got to admit it.”

I elbow him. Hard. “Very funny. Little Ms Awkward, eh?”

“Kat. You’re my number one cousin.”

“Who is destined to be an outsider.”

“What’s this?” he says, pointing to a footnote on the message. “Unknown risk of future mental health issues.”

I shrug. “No idea. Unless… Unless it’s linked to that weird time when they were all over us.”

He raises his bushy eyebrows. Not very attractive.

“Don’t pretend you don’t know. Mum used Gaite, the gateway app. Remember? To get an assessment for the school that was trying to fill its quotas for a diverse range of families? Got it now?”

He shakes his head.

How can he not remember? We were the focus of gossip for months among the uncles and aunts. Yes, I was only five years old, but I grew up with Mum and Dad’s stories about how hard it had been to get me into a decent primary school. Stories about the day the state came knocking. I had presumed that he’d heard them too.

“You must remember. Five minutes of Mum telling an app all about me kicked off an investigation.”

He looks at me as if I’m talking alien.

I shake my head. “They used our devices at home to monitor us and all the surveillance stuff out there to suck up as much data as they could to assess the family.”

He stares at me blankly. Not a clue.

“End result? The clever AI concluded that I was at risk of neglect. Oh, and it couldn’t even hint at any mental health issues I might develop as I grew up. That annoyed Mum and Dad. Too difficult for it to categorise me. Complex Kat, eh?”

He tuts. “Is that where that nickname came from? I had no idea.”

A big shout erupts from the other side of the garden. Yelling. Cheering. Clapping. Mum and Dad are getting ready to perform their party piece—a ballroom dance to an old-style track of electronic beats from their teenage days. It’s their speciality. Peculiarity, some say. The bass beat begins. The whoops increase and they take to the floor. I can’t bear to watch.

I can just about remember the nice woman who came to talk to us once a month. I remember my sister cursing after every visit and Mum and Dad being quieter than usual. My teachers often said how lucky I was to have the help and support to become better than my sister. That’s all very well, but am I being rejected because the recruitment algorithm can’t put me in a neat category that’ll help the police prove they have representative workforce? Or is it because of the footnote, the unknown risk of future mental health issues?

I turn my attention back to Malc, who is engrossed in the spectacle.

“Malc,” I shout above the music.

“I love your mum and dad. You’re so lucky,” he shouts back and continues watching in awe.

If only he knew how desperate I am to get Sofia away from this family and its destructive hedonism.

There’s a big cheer as they finish with a twirl and a bow. He turns his attention back to me.

“So, what happened?”

“With?”

“The whole ‘AI is watching you’ thing.”

“Yeah, in the end they reckoned there was a glitch in the training data. Well, not a glitch exactly. More of a chasm.”

“Do tell.”

“They realised there was a deep-seated bias against certain lifestyles. All a bit ‘soap-opera morality gone wrong’.”

“Typical.”

“Yeah. They fixed it though. Retrained the blinking thing and then reassessed us when my sister went bad. Malc, I can’t believe you don’t know this stuff.”

He blushes. “I never really told you, did I? When I was a kid, Mum and Dad didn’t want to talk about your family. ‘An embarrassment’ was all they’d say.”

“Bloody hell,” I say, rubbing my temples. “Not surprising, I guess.”

“So they retrained it?”

“Yeah. Second time around, no problems noted. And I was diagnosed with a bunch of stuff they reckoned only a human could have worked out.”

“Complex Kat.”

“Yeah. Complex Kat.”

He fills his cheeks with air, holds it for a few moments, and then lets it out with a loud sigh.

“Why is that still there then?” he says, pointing at the footnote of the message.

I scratch the back of my neck and fiddle with my hair. An annoying habit.

“Good point,” I say.

I scroll the message up and down to see if there might be some hidden caveat or a piece of text I’ve missed. Nothing.

We watch the dancing. The music booms, the bodies sway, and the drinks are flowing. It’s my birthday and they’ve forgotten me. Not that I mind. I didn’t want the party in the first place. The last thing I want is to be the centre of attention. I’m eighteen and I want to be a police officer. All I want— Wait—

How can I have been so stupid?

“Oh my—” I say. “Guess what? You know I said they redid us when I was twelve?”

He nods.

“I refused to let the data be used in any way. I was being bullied. For being different. I was so freaked out, I made sure all of it—on me, my mum, my dad—was totally secure. No ammo for the bastards who were making my life a misery.”

“So?”

“Yeah. Don’t you get it? Unknown issues. The police don’t know. I was diagnosed. They could place me in one of their diversity categories. Help with their quotas. Let me—”

I quickly bring up my privacy app and scroll until I find it. I select the option to allow public bodies to access the data. Almost instantaneously another message from the application app arrives. I’ve been approved for the next stage of interview. I’m on my way. I give Malc a big smacker of a kiss on the cheek, hand my precious baby to him, and run across the yard to join Mum and Dad on the dance floor.

Sofia’s going to be fine.
